# Spatial-Controlled Coating of Pro-Angiogenic Proteins on 3D Porous Hydrogels Guides Endothelial Cell Behavior

**DOI:** 10.3390/ijms232314604

**Published:** 2022-11-23

**Authors:** Chau Le Bao, Helen Waller, Alessandra Dellaquila, Daniel Peters, Jeremy Lakey, Frédéric Chaubet, Teresa Simon-Yarza

**Affiliations:** 1 Laboratory for Vascular Translational Science (LVTS) INSERM U1148, Université Paris Cité, Université Sorbonne Paris Nord, CEDEX 18, 75877 Paris, France; 2 Biosciences Institute, Newcastle University Biosciences Institute, Newcastle upon Tyne NE1 7RU, UK

**Keywords:** hydrogels, electrostatic interactions, spatial-controlled coating, angiogenesis, tissue engineering

## Abstract

In tissue engineering, the composition and the structural arrangement of molecular components within the extracellular matrix (ECM) determine the physical and biochemical features of a scaffold, which consequently modulate cell behavior and function. The microenvironment of the ECM plays a fundamental role in regulating angiogenesis. Numerous strategies in tissue engineering have attempted to control the spatial cues mimicking in vivo angiogenesis by using simplified systems. The aim of this study was to develop 3D porous crosslinked hydrogels with different spatial presentation of pro-angiogenic molecules to guide endothelial cell (EC) behavior. Hydrogels with pores and preformed microchannels were made with pharmaceutical-grade pullulan and dextran and functionalized with novel pro-angiogenic protein polymers (Caf1-YIGSR and Caf1-VEGF). Hydrogel functionalization was achieved by electrostatic interactions via incorporation of diethylaminoethyl (DEAE)–dextran. Spatial-controlled coating of hydrogels was realized through a combination of freeze-drying and physical absorption with Caf1 molecules. Cells in functionalized scaffolds survived, adhered, and proliferated over seven days. When incorporated alone, Caf1-YIGSR mainly induced cell adhesion and proliferation, whereas Caf1-VEGF promoted cell migration and sprouting. Most importantly, directed cell migration required the presence of both proteins in the microchannel and in the pores, highlighting the need for an adhesive substrate provided by Caf1-YIGSR for Caf1-VEGF to be effective. This study demonstrates the ability to guide EC behavior through spatial control of pro-angiogenic cues for the study of pro-angiogenic signals in 3D and to develop pro-angiogenic implantable materials.

## 1. Introduction

Tissue engineering has offered the tantalizing possibility to regenerate tissues and organs, allowing the treatment of a multitude of conditions and pathologies. Despite numerous significant progresses with in vitro and small animal studies, clinical applications have been scarce [1]. Even the most advanced solutions delivered to physicians lack sufficient vascularization within the tissue engineered constructs [2]. This is because the diffusion of oxygen and nutrient supply present major limits on the size and complexity of bioengineered scaffolds. For this reason, vascularization of biomaterials remains the highlighted focus in tissue engineering and regenerative medicines. In this context, one main current challenge in tissue engineering is the development of biomaterials that can promote angiogenesis, ultimately integrating with the host vasculature to form anastomosis.

Angiogenesis, the formation of new blood vessels from existing ones, is a complex process. During angiogenesis, quiescent endothelial cells (ECs) from pre-existing vessels are activated by the increase in concentration of pro-angiogenic factors induced by inflammation or by hypoxia [3]. Activated ECs proliferate and differentiate into tip cells, which results in the elongation of new blood vessels in the direction of the pro-angiogenic stimulus. This sprouting process is modulated by the migration of ECs led by tip cells, characterized by lamellipodia and filopodia in their cytoskeleton, followed by stalk cells, which are found between quiescent cells and the tip cells. Stalk cells continue to proliferate and constitute the new endothelium, while ensuring a continuum with the original vessel through regulated proliferation [4,5]. Once the capillary is formed, ECs secrete attractant molecules to recruit perivascular cells, which migrate along the newly formed vessels and provide stability, support cell differentiation, and regulate vessel permeability [6].

Angiogenesis is partially modulated by the ECM, which provides essential structural support and biochemical cues for cell morphogenesis and physiological functions [7]. Numerous strategies employing hydrogels with functionalized pro-angiogenic molecules have been proposed to promote vessel formation. Most of these approaches are based on the delivery of growth factors (GFs), such as vascular endothelial growth factor (VEGF), to facilitate vascularization in vivo [8]. Recently, pre-vascularization of biomaterials has been proposed as an approach to promote in vitro vessel formation prior to implantation. The idea is to stimulate in vitro vessel formation within 3D biomaterials which present pre-formed channels. Different techniques to develop hydrogels with pre-formed channels have been investigated. These include the use of syringes or glass micropipettes [9], or sacrificial templates [10,11,12,13]. To promote cell adhesion, ECM proteins (e.g., collagen, fibrin, or fibronectin) and cell adhesive molecules (e.g., RGD, YIGSR sequences) are often incorporated into the hydrogel composition [8,14,15]. Besides interaction with the ECM, angiogenesis also depends on spatial presentation of pro-angiogenic cues that direct vessel sprouting and maturation [3]. Over the past decades, various approaches have attempted to fabricate hydrogels with spatial guidance either through direct patterning of vascular cells, or through spatial distribution of pro-angiogenic molecules (e.g., VEGF, FGF, angiopoietin, YIGSR) [16,17,18,19,20]. The use of ECM molecules presents promising outcomes for in vitro and in vivo vascularization. Nevertheless, clinical translation still remains a hurdle due to high cost and immunogenic potential of animal-origin ECM molecules.

Several important factors must be taken into account when designing hydrogels that favor endothelialization for tissue engineering and regenerative medicines: (1) presence of interconnected pores favoring cell–cell interactions and migration; (2) presence of a hollow channel having a wide range of diameters to mimic native vessels; (3) ability to promote EC arrangement leading to the formation of microvessel-like networks; (4) biocompatible composition (pharmaceutical-grade materials); (5) integration of basement membrane proteins (BM), such as laminin and collagen type IV, and other ECM proteins to induce endothelial proliferation and differentiation during angiogenesis; (6) easy fabrication protocol; and (7) cost efficient.

For vascularization purposes, porous 3D hydrogels are widely employed due to their ability to facilitate nutrient and oxygen diffusion, thus enabling cell migration [21,22]. Additionally, the presence of channels within porous scaffolds has been reported to promote cell growth and rapid vascularization [23,24]. The channels in 3D hydrogels play a key role in guiding EC arrangement and should also be utilized to induce angiogenic behavior in ECs.

Polysaccharides are widely employed as tissue engineered biomaterials due to their physicochemical properties that can mimic the ECM [25]. In this context, we utilized 3D porous hydrogels, composed of pullulan and dextran. Notably, our team has demonstrated in several studies the versatility of pullulan- and dextran-based hydrogels, where the scaffold geometry, mechanical properties, porosity, and swelling behavior of these hydrogels could be controlled [25,26,27,28]. The hydrogel crosslinking method was previously described in numerous publications and has been patented [29,30]. Thus, these hydrogels have been investigated in various in vitro and in vivo studies [29,31,32]. Most recently, we have demonstrated the ability to guide EC arrangement based on channel curvature on the 3D polysaccharide hydrogels [28].

**Figure 1:**
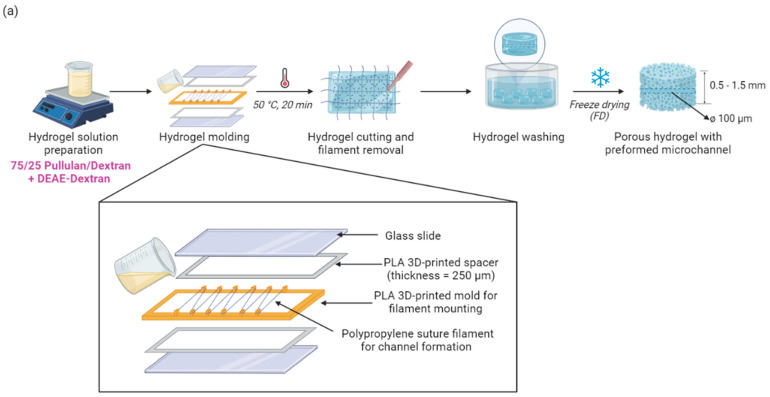

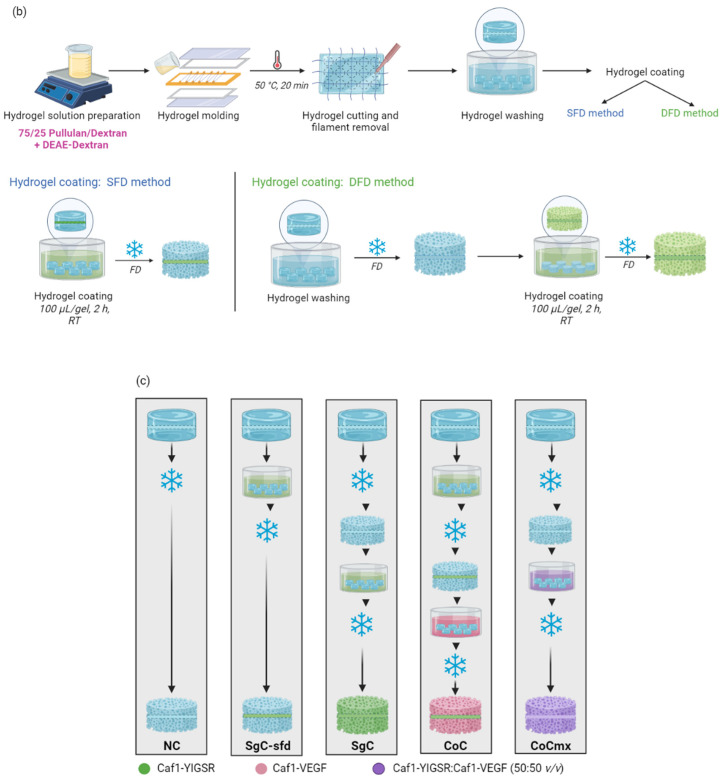
(**a**) Fabrication protocol of 3D porous hydrogels without coating; (**b**) Fabrication protocol of 3D porous hydrogels with coating (SFD: single freeze-drying; DFD: double freeze-drying); (**c**) Schematic plan of spatially controlled coating methods. NC: non-coated; SgC-sfd: single-coated-single-freeze-drying; SgC: single-coated; CoC: Co-coated; CoCmix: co-coated-co-mixed.

In the context of promoting in vitro vessel formation, this study aimed to develop 3D porous hydrogels with different spatial presentation of pro-angiogenic signals to guide ECs towards angiogenic behavior. The challenge of this work was to functionalize the chemically crosslinked hydrogels to promote EC adhesion and to direct sprouting through spatial guidance using pro-angiogenic cues. Here, we present a simple method to produce biomimetic 3D porous hydrogels, made from pharmaceutical-grade pullulan and dextran, with preformed microchannels (Figure 1). To provide cells with pro-adhesive and pro-angiogenic signals, the hydrogels were functionalized using a recombinant, engineered bacterial protein polymer called Caf1. Caf1 subunits assemble into long, highly stable and flexible polymers, which are bioinert, allowing bioactive peptide motifs from the ECM and growth factors to be inserted and hence provide exquisite control over the biological signals supplied to the cells [33,34,35]. In this work, we demonstrate an innovative strategy to functionalize chemical hydrogels in a spatial-controlled manner. Capitalizing on the acidic pI of Caf1, we could functionalize hydrogels simply via electrostatic interactions induced by the coating method (Figure 1b). Then, spatial cues of the pro-angiogenic motifs were modulated through a combination of hydrogel coating and a freeze-drying process (Figure 1c).

The developed scaffolds were evaluated based on: (1) porosity; (2) presence of the hollow channels formed within the 3D scaffolds; (3) ability to promote EC cell adhesion as well as migration; (4) ability to induce pro-angiogenic behavior of ECs. Furthermore, our approach offers a facile protocol for both scaffold fabrication and functionalization. The use of Caf1 overcomes the high cost and immunogenic potential of traditional ECM molecules. The functionalized scaffolds exhibited good EC adhesion and proliferation. Scaffolds with different spatial distribution of pro-angiogenic moieties induced different EC behaviors. Based on the results obtained from this study, we report the first work, to our knowledge, in using animal-free ECM-like molecules to control the spatial cues of hydrogel-based scaffolds, which modulates EC behavior and guides them towards angiogenic sprouting.

## 2. Results

### 2.1. Hydrogel Preparation and Characterization

**Figure 2:**
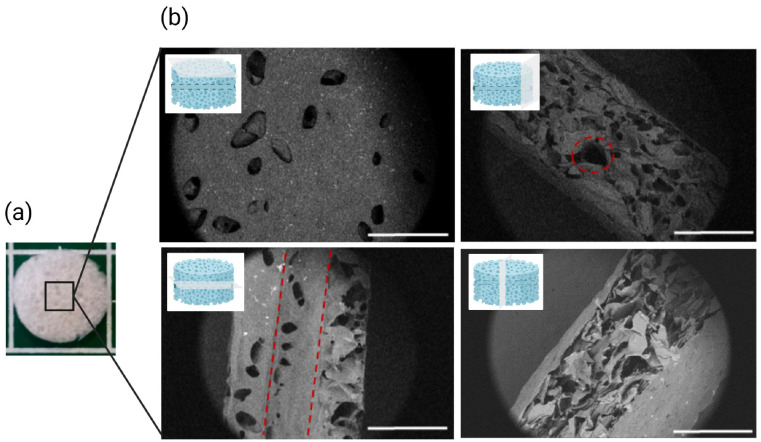
(**a**) Image of the hydrogel showing pores visible to the naked eyes. Scale bar = 5 mm; (**b**) SEM images of the surface, the edges and the cross-section of the hydrogel with a preformed channel. Red dash circle shows the circular cross-section of the microchannel (ø~100 µm), observed on the side of the hydrogel. Red dashed lines represent the limit between the hollow channel and the hydrogel surface. Scale bar = 1 mm.

Hydrogels molded using spacers and cut into discs had an average thickness of 550 ± 20 µm after freeze-drying. Surface pores were clearly visible with the naked eye (Figure 2a). Scanning electron microscopy (SEM) observations confirmed the porous structure, revealing the macro- and micro-architecture of the hydrogels (Figure 2b). Larger pores (>50 µm) were present on the surface, while interconnected smaller pores (<50 µm) were seen in the cross-section of these hydrogels. Additionally, the presence of a hollow channel in the middle of the hydrogel was observed along with pores inside the channel structure (Figure 2b, bottom left).

**Figure 3:**
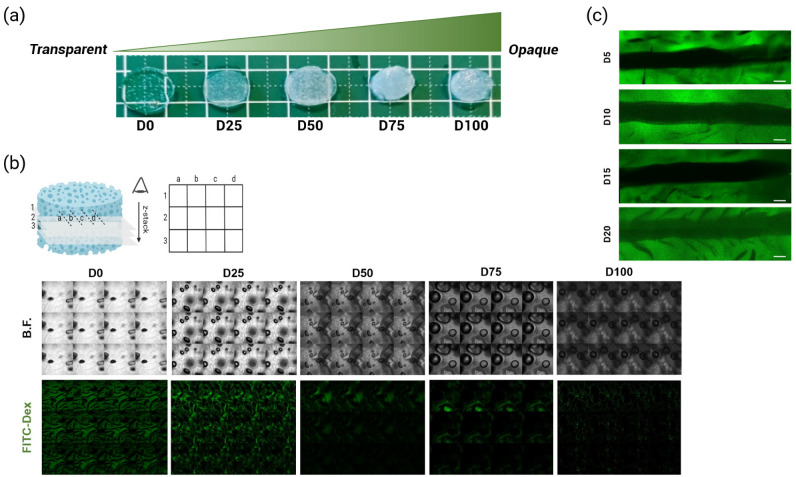
(**a**) Hydrogel opacity increased with an increase in DD concentration; (**b**) Hydrogel opacity as observed using CLSM: under bright-field and fluorescence (FITC). Z-stack images of hydrogels without DD (D0) and with DD 25–100% (DD:Dex *w*/*w*) were compiled as collages to demonstrate the increase in sample opacity with an increase in sample depth. (**c**) Z-projection (average intensity) of FITC-Dex hydrogels observed using CLSM.

The cationization by DEAE–Dex (DD) on pullulan–dextran-based hydrogels, previously described by our team [25,26,27,28] (referred to as PUDNA), was proven by an increase in the zeta potential of the hydrogel (from −22.3 mV to +8.28 mV) when replacing dextran with DD. Based on these results, a series of experiments were carried out to determine the optimal concentration of DD needed to facilitate electrostatic interactions between the cationized hydrogel and the negatively charged Caf1 protein polymer. Hydrogel solutions with various DD concentrations were prepared (25%, 50%, 75%, and 100% DD:Dex *w*/*w*) and hydrogels were formulated following the protocol described, as shown in Figure 1. These hydrogels were referred to as D0 for non-cationized samples, and D25, D50, D75, D100 for cationized samples with varying DD concentrations aforementioned. Upon rehydration of the hydrogels for further characterization, it was observed that the opacity increased with the increase in DD concentration added to the hydrogel (Figure 3a). Between D25 and D50, the samples were already quite opaque but the structures next to the surfaces were still visible under the microscope (Figure 3b). However, above D25, the gels were too opaque to allow observation, using confocal laser scanning microscopy (CLSM) or biphoton microscopy, of the microchannel which was embedded in the middle of the hydrogel (*z*-axis). Therefore, another opacity-coating efficiency test was conducted at the lower range of DD concentration: 5%, 10%, 15%, and 20% (DD:Dex *w*/*w*) (Figure 3). Here, all the hydrogels and their channel structures were visible via the confocal microscope (Figure 3c). Thus, all four conditions (D5, D10, D15, D20) were used for further hydrogel characterization as well as for in vitro studies with ECs.

Porosity measurements of non-cationized (PUDNA) and cationized hydrogels containing DEAE-dextran (PUDNA-D5, PUDNA-D10, PUDNA-D15, and PUDNA-D20) showed porosity values of 25–28% (Table 1). Swelling ratios for all conditions were around 12 (Table 1) meaning that water content after swelling was around 93 % (*w*/*w*). It should be noted that we did not find statistical differences between the different formulations.

The degree of crosslinking by sodium trimetaphosphate within the hydrogels was evaluated by quantifying the amount of phosphorus present after matrix degradation with HNO_3_, an indicator of phosphate bridges between chains of pullulan–dextran. The phosphorus content in all hydrogel conditions ranged from 113 to 143 µmol per gram of hydrogel. These results suggest that the incorporation of DEAE–dextran did not affect the crosslinking degree of the polysaccharide-based hydrogels.

### 2.2. In Vitro Endothelial Cell Studies

To ensure cell adhesion on the materials, Caf1-YIGSR (a Caf1 polymer containing a pro-adhesive peptide sequence from laminin) was used to functionalize the hydrogels via electrostatic interactions. First, hydrogels were cationized by incorporating DD at various concentrations (5–20% DD:Dex *w*/*w*). Then, the cationized scaffolds were coated with the solution of Caf1-YIGSR (1 mg/mL, p.I. = 4.6) [13], at pH 7.0, room temperature (RT) via the vacuum-induced syringe method (Figure S1). This technique ensured that only the channel was coated. Subsequently, after the syringe coating step, the scaffolds were submerged in the same Caf1-YIGSR solution for 2 h, RT and immediately rinsed with PBS before the freeze-drying step (Figure 1b, SFD method). The scaffolds were exposed to UV light for at least 1 h before cell seeding experiments.

#### 2.2.1. Selection of Cationized Hydrogel for Optimal Coating Efficiency

To establish the optimal concentration of DD required to functionalize the scaffolds via electrostatic interactions, samples with increasing DD concentration (5–20% DD:Dex *w*/*w*) were coated, then loaded with HUVECs at a seeding density of 5.0 × 10^6^ cells/mL. Coating efficiency was determined based on cell adhesion and cell morphology. After 7 days in culture, cellularized scaffolds were fixed and stained with DAPI and phalloidin TRITC. Coated, cationized scaffolds with 5–20% DD:Dex (*w*/*w*) were referred to as PUDNA-D5C, PUDNA-D10C, PUDNA-D15C, and PUDNA-D20C, respectively.

**Figure 4:**
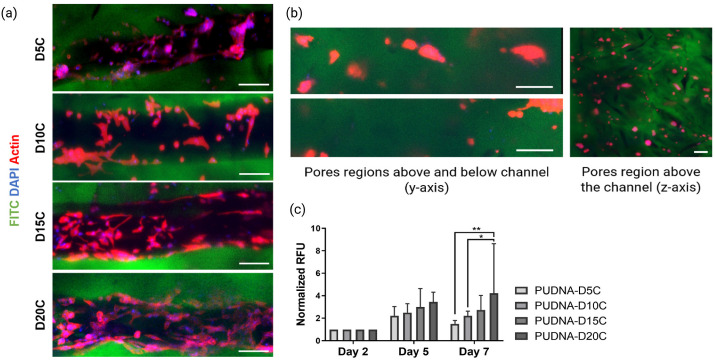
Cell analysis of seeded HUVECs on coated, cationized hydrogels with increasing DD concentrations: (**a**) Images represent Z-Projection, average intensity, showing cell morphology at day 7 via CLSM. Scale bar = 100 µm; (**b**) Representative image (Z-Projection) of cells in the pore region outside the channel on coated scaffold. Scale bar = 100 µm; (**c**) Cell metabolic activity determined by resazurin assay on days 2, 5, and 7. All resofurin fluorescence unit (RFU) values of each condition were normalized to their own RFU value on day 2. Statistical analysis was performed using a two-way ANOVA with multiple comparisons. * *p* < 0.05, ** *p* < 0.01.

HUVECs seeded on PUDNA-D5C formed large aggregates inside the coated channel section (Figure 4a). On PUDNA-D10C, a few polarized cells could be detected, where they exhibited filopodia and connections to neighboring cells (Figure 4a). On PUDNA-D15C scaffolds, the number of cells that adhered inside the channel appeared to increase slightly. The cell clusters seemed to reduce, while more polarized cells appeared inside the channel. Finally, on PUDNA-D20C scaffolds, cell morphology and behavior significantly improved. The entire channel edge was lined with elongated cells. These cells formed connections with their neighboring cells, showing filopodia structure and stress fibers, and less cell aggregates were detected. Regarding the porous regions outside the channel structure, numerous cell clusters were observed in the pores neighboring the channel (*y*-axis) as well as in the macropores outside the channel (*z*-axis) (Figure 4b).

Cell metabolic activity on all coated, cationized scaffolds was also investigated (Figure 4c). Overtime, there was an increase in cell metabolic activity for all coating conditions. On day 7, metabolic activity reached its peak for all conditions, with PUDNA-D20C showing the highest value and statistically greater than the metabolic activity on PUDNA-D5C and PUDNA-D10C. Although the cell metabolic activity on PUDNA-D15C vs. PUDNA-D20C did not differ, the morphological organization of HUVECs on PUDNA-D20C appeared more superior to those on PUDNA-D15C. From here on, PUDNA-D20C which showed optimal coating efficiency, was chosen as the standard cationized hydrogel for future functionalization experiments.

#### 2.2.2. Spatial-Controlled Coating: Caf1-YIGSR Facilitated Cell Adhesion in Both SFD and DFD Coating Methods

We hypothesized that the coating of hydrogels could be modulated by integrating the coating step (via vacuum-induced syringe method) before and after the freeze-drying (FD) step (Figure 1). To confirm this hypothesis, cationized hydrogels (PUDNA-D20) were functionalized twice: the first coating was performed before FD, then the second coating was performed after FD. Samples which were coated once, were only freeze-dried once, in which only the channel was coated. These samples were named SFD and were used as controls. On the other hand, samples that were coated twice, hence freeze-dried twice, were named DFD, in which both the channel and the pores were coated.

**Figure 5:**
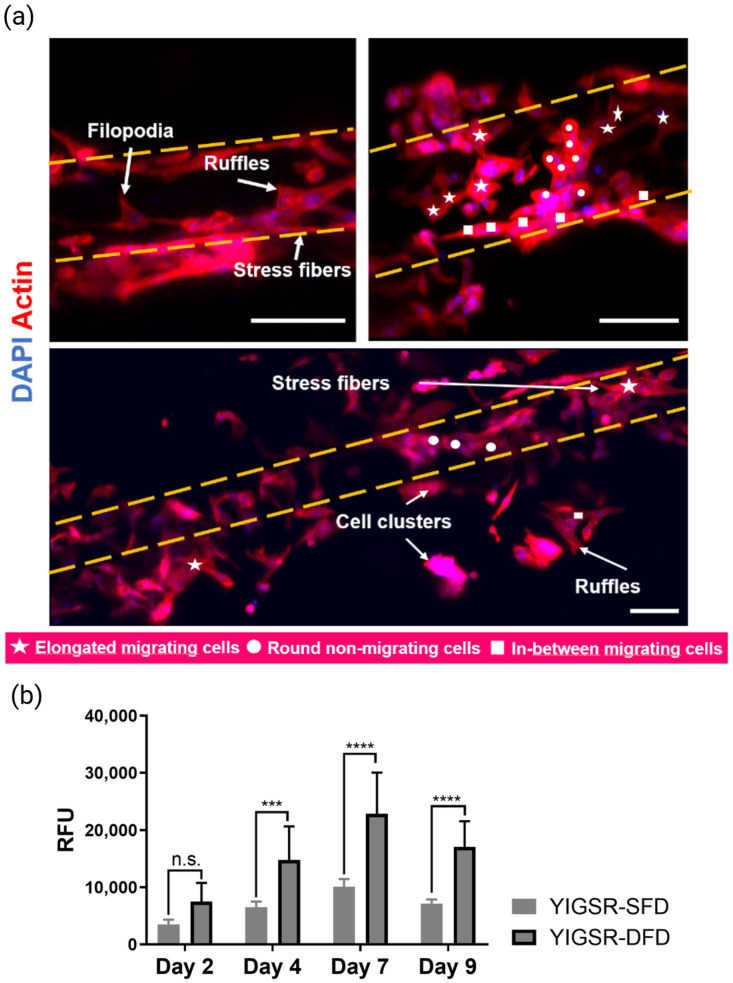
Cell analysis of seeded HUVECs on PUDNA-D20C scaffolds (SgC-SFD, top; SgC-DFD, bottom). (**a**) Images represent Z-Projection (average intensity) showing cell morphology at day 7 via CLSM. Scale bar = 100 µm; (**b**) Cell metabolic activity determined by resazurin assay on days 2, 5, 7, and 9. Statistical analysis was performed using a two-way ANOVA. *** *p* < 0.001, **** *p* < 0.0001.

The coated hydrogels were seeded with HUVECs at 5.0 × 10^6^ cells/mL and cultured for 9 days. Then, cellularized scaffolds were analyzed for cell adhesion, cell morphology, and cell metabolic activity. Similar to the SFD scaffolds, cells on the DFD scaffolds adhered in a monolayer along the channel lining and more cell spreading (elongation) was detected after 7 days in culture. Additionally, more polarized cells were observed inside the channel (Figure 5a, bottom). When looking at the pores near the channel edges, migrating cells were observed: the cell filopodia reached towards the pores outside the channel and formed connections with neighboring cells residing in the pores (external of channel) (Figure 5a, bottom). Cell clusters were also detected: inside the channel, the clusters were comprised of both polarized and round cells; outside the channel, the clusters were composed of mostly round cells. On day 9 (results not shown), the cell’s presence began to block visibility under the CLSM, making it inconclusive for further analysis.

Cell metabolic activity from resazurin assay (Figure 5b) was analyzed to support the cell adhesion and cell morphology observations. For both SFD and DFD scaffolds (YIGSR-SFD and YIGSR-DFD), there was an increase of metabolic activity from day 2 to day 7. After 7 days, the metabolic activity dropped. Compared to SFD, cells on DFD scaffolds had a significantly higher metabolic activity, with a peak on day 7.

#### 2.2.3. Spatial-Controlled Coating (DFD Method): Caf1-YIGSR and Caf1-VEGF Influenced Cell Behavior Differently

**Figure 6:**
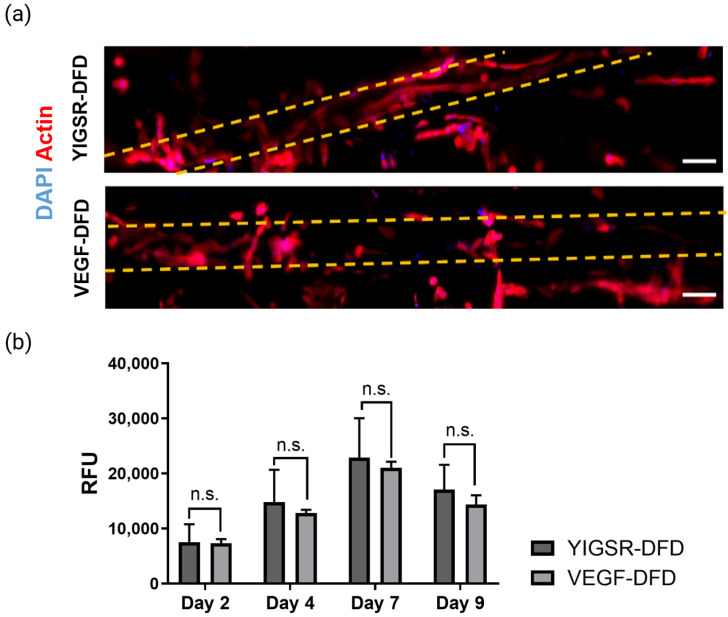
Cell analysis of seeded HUVECs on scaffolds functionalized with Caf1-YIGSR and Caf1-VEGF via the DFD method. (**a**) Tile-scan images represent Z-Projections (average intensity) showing cell morphology inside the scaffold channel (full length) at day 7 via CLSM. Scale bar = 100 µm. Yellow dashed lines represent the limit of the microchannel; (**b**) Cell metabolic activity of seeded HUVECs determined by resazurin assay on days 2, 4, 7, and 9. Statistical analysis was performed using a two-way ANOVA.

Two different recombinant Caf1 proteins containing peptide sequences from laminin (YIGSR) and VEGF, were tested on DFD hydrogels. Those coated with Caf1-YIGSR and with Caf1-VEGF, were named YIGSR-DFD and VEGF-DFD, respectively. Regarding the cell morphology, cells seeded on hydrogels coated with YIGSR exhibited different shape than those seeded on VEGF-coated hydrogels (Figure 6a). On VEGF-DFD samples, very few cells adhered inside the channel and the channel edge. Those that remained adhered inside the channel started to polarize. In contrast, on YIGSR-DFD samples, a greater number of cells adhered inside the channel and lined the channel edge while fewer cells showed signs of migration or polarization.

Overtime, cell metabolic activity followed the previously observed trend, with the highest cellular activity observed on day 7 and a slight decrease on day 9. The differences in cellular activity of seeded HUVECs on YIGSR-DFD and VEGF-DFD were insignificant (Figure 6b), but the variations of cell morphology seen on the differently coated scaffolds were more obvious (Figure 6a). These results confirmed that Caf1-YIGSR had a stronger cell-adhesive effect than Caf1-VEGF.

#### 2.2.4. Spatial-Controlled Coating (SgC, CoC, CoCmx): Caf1-YIGSR and Caf1-VEGF Spatial Distribution on Scaffolds Can Provoke Different Angiogenic Behaviors

Using the optimal coating protocol (DFD = double freeze-drying, coating of both channel and pores), the next step was to determine whether (i) the presence of different protein types (Caf1-YIGSR and Caf1-VEGF) and (ii) their spatial presentation on the scaffolds (pores and/or microchannel) could influence cell behavior. To answer this question, several coatings were performed as described in Figure 1c. DFD scaffolds with only one type of coating (Caf1-YIGSR) were named SgC. Scaffolds with two types of coating (Caf1-YIGSR and Caf1-VEGF) were named CoC and CoCmx. In CoC, Caf1-YIGSR were coated only in the channel and Caf1-VEGF were coated in the pores. In CoCmx, both Caf1 sequences were mixed at a 50:50 ratio (*v*/*v*) so that the pores and the channel were simultaneously coated at the same time with both Caf1-YIGSR and Caf1-VEGF (Figure 4). The non-coated, cationized hydrogels (D20C) were used as control and were named NC.

**Figure 7:**
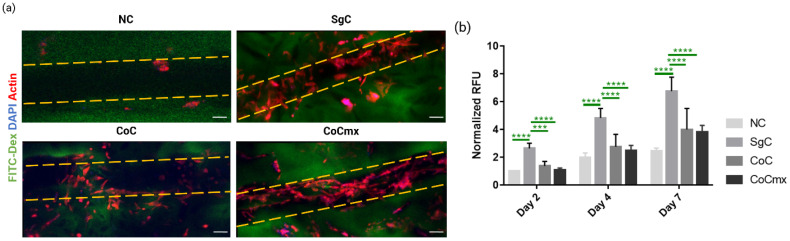
Cell analysis of seeded HUVECs on non-functionalized (NC) and functionalized scaffolds with a different spatial distribution of Caf1-YIGSR and Caf1-VEGF (SgC, CoC, and CoCmx) via the DFD method. (**a**) Images represent Z-Projection (average intensity) showing cell morphology at day 7 via CLSM. Scale bar = 100 µm. Yellow dashed lines represent the limit of the microchannel; (**b**) Cell metabolic activity (resazurin-based assay) at days 2, 4, and 7. Statistical analysis using two-way ANOVA of all hydrogels compared to SgC. Statistical analysis was performed using a two-way ANOVA. *** *p* < 0.001, **** *p* < 0.0001.

**Figure 8:**
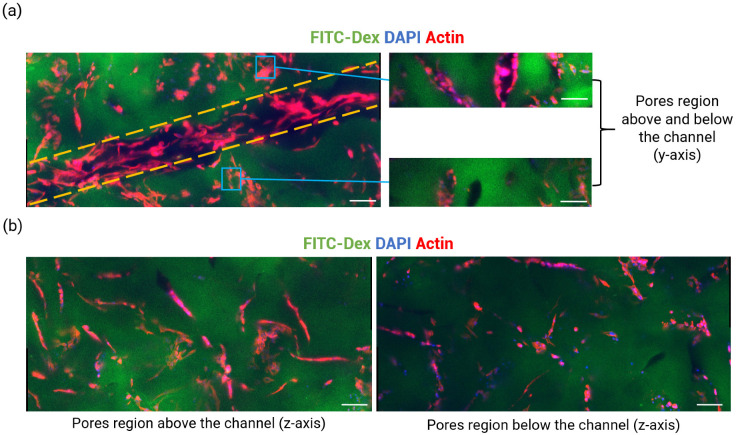
Cell analysis of seeded HUVECs on CoCmx scaffolds. (**a**) Images represent Z-Projection (average intensity) inside the scaffold’s channel. Scale bar = 100 µm. Yellow dashed lines represent the limit of the microchannel; (**b**) Images represent Z-Projection (average intensity) of the same scaffold, in the porous regions outside the channel (*z*-axis). Scale bar = 100 µm.

As expected, the NC scaffolds did not support cell adhesion or proliferation overtime (Figure 7). On the NC scaffolds, only cell aggregates were observed inside the channel and cell metabolic activity was lower compared to those on the coated scaffolds. Initial examination of cell morphology on all the coated scaffolds (SgC, CoC, CoCmx) showed interesting outcomes. Both SgC and CoCmx resulted in elongated cells lining the channel edges, a high density of polarized cells inside the channel, large number of cells forming connections with neighboring cells. While both CoC and CoCmx scaffolds encouraged adhered cells to migrate outwards of the channel, SgC scaffolds only contained adhered cells within the channel (Figure 7a). Further observations on CoCmx scaffolds demonstrated that the co-presence of Caf1-YIGSR and Caf1-VEGF also had an effect on ECs outside the channel (Figure 8). Here, the cells in the pores did not form aggregates but rather exhibited an elongated morphology, conforming their shape to the curvature of the pores (Figure 8a, right panel and Figure 8b).

Overall cell metabolic activity showed the expected trend with the highest activity observed on day 7 for all scaffold conditions. NC scaffolds resulted in the lowest cell metabolic activity, which is representative of the cell morphology outcome. As for coated scaffolds, the cell metabolic activity on SgC samples was significantly greater compared to those on the CoC and CoCmx scaffolds (Figure 7b).

#### 2.2.5. Protein Bulk Concentration

Previously, we observed that the cell metabolic activity of HUVECs seeded on all functionalized hydrogels was statistically higher than on non-functionalized ones. Moreover, cell morphology drastically improved when seeded on spatially controlled coated DFD gels (SgC, CoC, CoCmx) (Figure 7a). Therefore, we hypothesized that the enhancement in cell behaviors was contributed by an increase in protein concentration on the functionalized scaffolds.

**Figure 9:**
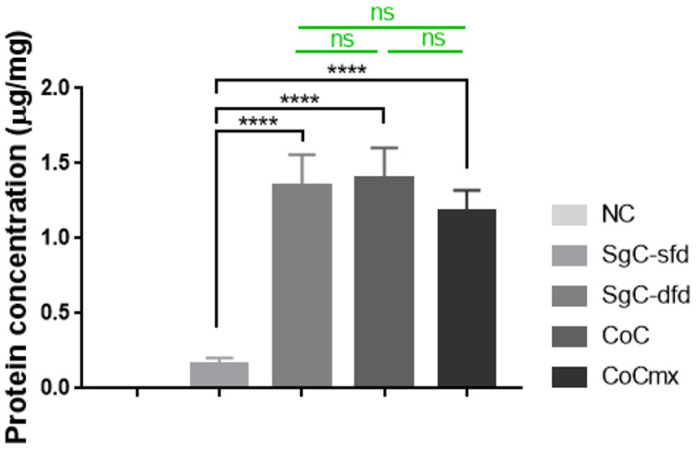
Protein concentration of cationized hydrogels (20% DEAE–Dextran) with spatial-control coating. Statistical analysis was performed using a one-way ANOVA. **** *p* < 0.0001.

Among the SgC scaffolds where only one type of protein (Caf1-YIGSR) was employed and the coating was performed either once (SFD, channel coated only) or twice (DFD, channel and pores coated), the DFD scaffolds had a significantly greater protein concentration (µg per mg hydrogel). Similarly, when comparing all the DFD samples to the SgC-SFD samples, CoC and CoCmx showed a higher protein concentration (Figure 9). These values were expected since the DFD scaffolds were coated twice, hence the number of proteins grafted onto the hydrogels would be greater on these scaffolds.

## 3. Discussion

Porous hydrogels made of pullulan and dextran were synthesized by chemical crosslinking with sodium trimetaphosphate (STMP), as previously described [29]. First, the hydroxyl groups in the polysaccharides were activated at basic pH using NaOH, resulting in the opening of cyclic STMP and crosslinking between the polysaccharides, leading to hydrogel formation [36]. Previous studies have demonstrated the potential of these biocompatible hydrogels as scaffolds for 3D cell culture, tissue engineering, and cell therapy applications [25,27,37,38]. However, due to the high water content (~93%) and the chemical structures of pullulan and dextran, endothelial cells do not adhere spontaneously to these hydrogels [28,38], as shown in Figure 7a (NC sample). The neutral polysaccharides were cationized by incorporating diethylaminoethyl dextran (DEAE–Dex) to facilitate electrostatic interactions with the negatively-charged ECM-like molecules (Caf1-YIGSR and Caf1-VEGF). The shape and diameter of the channel remained 100 ± 20 µm before and after swelling. These observations are in correspondence with the swelling behavior observed in all formulations. Here, we demonstrated the ability to form straight microchannels with circular cross-section and controlled diameter.

Cell analysis of HUVECs seeded on functionalized scaffolds with various coating methods (SFD vs. DFD) and spatial distribution of the two Caf1 motifs (YIGSR and VEGF) confirmed that the Caf1 solution (p.I. = 4.6; 1 mg/mL; ɀ = −23.6 mV) was adequate to facilitate electrostatic interactions with cationized hydrogels (PUDNA-D20; ɀ = +29.5 mV). Through the addition of microchannels within the polysaccharide scaffolds and sufficient surface functionalization via electrostatic interactions, ECs were able to adhere, leading to good cell proliferation and cell spreading within the microchannel. In this work, spatial control of the ECM-mimicking moieties was shown to induce different EC behaviors that could be interesting for vascularization applications. An in-depth discussion on this part is presented in Section 3.2, Section 3.3 and Section 3.4.

### 3.1. The Impact of DEAE–Dex Concentration on Hydrogel Opacity and Functionalization

As seen in Figure 3, an increase in the concentration of DEAE–Dex added to the polysaccharide solution contributed an increase in hydrogel opacity, which limited sample visibility under the microscope. Even with a multiphoton microscope or a high-resolution confocal laser scanning microscope and sufficient image treatment and analysis, it was very difficult to locate the microchannel embedded in the middle of the sample depth. Consequently, these observations suggest that a balance between hydrogel transparency and the cationic polymer concentration need to be considered to ensure sample visibility for microscopy analysis, which is essential to monitor cell behavior in the scaffolds. Moreover, this balance must also allow sufficient interactions between the charged materials in order to facilitate cell adhesion on the functionalized scaffolds.

After a series of optimization work, by synthesizing cationized hydrogels with varying DEAE–Dex concentrations (5–100% DD:Dex *w*/*w*), we were able to determine the best cationic parameters to yield optimal hydrogel opacity and favorable EC behavior outcomes. At 20% (DD:Dex *w*/*w*), the scaffold surface and microchannel were still visible under the CLSM (Figure 3a). More importantly, ECs were also observable after 7 days in culture (Figure 4a). On day 7, PUDNA-D20C scaffolds facilitated better EC adhesion, where more elongated cells were present inside the channel (Figure 4a) and cell metabolic activity was statistically higher than the metabolic activity on the rest of the other conditions (Figure 4c). These results strongly suggest that PUDNA-D20C was the optimal hydrogel condition for most favorable EC adhesion and morphology.

### 3.2. Caf1-YIGSR Induced Cell Adhesion on SFD Hydrogels and Enhanced Cell Proliferation on DFD Hydrogels

Cell morphology and behavior on SFD scaffolds using Caf1-YIGSR (Figure 5a) confirmed that the YIGSR sequence could be used as a cell-adhesive coating material.

Recent works on functionalized biomaterials have demonstrated the ability to modulate cell behavior by varying the concentration of cell-adhesive ligands in the scaffolds, with an increase in ligand concentration leading to an improvement in cell adhesion, spreading, and proliferation [39]. The results obtained from this study are in accordance with these findings. When both the channel and pores were coated (DFD coating method: YIGSR-DFD), the amount of Caf1 protein detected on the SgC-DFD scaffolds was higher than that on the SgC-DFD scaffolds, suggesting an increase in bulk ligand concentration (Figure 9). As a result, cell morphology on the DFD scaffolds greatly improved (Figure 5a) and greater cell metabolic activity was observed after 7 days in culture (Figure 5b). The decrease in cell metabolic activity on day 9 could be due to high cell confluency. This is supported by the fact that no signs of cell death were observed after 9 days. In conclusion, the increase in spatial distribution of the YIGSR sequence, contributed to an increase in ligand bulk concentration on the scaffold, leading to an enhancement of EC morphology and behavior.

### 3.3. Caf1-VEGF Induced Cell Migration and Angiogenic Sprouting Depending on Its Spatial Presentation on Porous Hydrogels

When Caf1-VEGF was used alone as the coating material in the DFD method, few cells adhered inside the channel and did not completely line the channel edge. Adhered cells showed polarization characteristics and sprouting-like behaviors. It is well known that VEGF is a pro-migratory factor that induces filopodia elongation in ECs during angiogenesis [5]. This explains why ECs in VEGF-DFD scaffolds showed filopodia structure resembling migration behaviors (Figure 5a). Additionally, cells on the top side of the channel protruded and connected to cells on the bottom side (Figure 6a). Due to lack of cell-adhesive moieties (i.e., Caf1-YIGSR), not enough cells adhered inside the channel, resulting in lower cell proliferation, as demonstrated by the lower cell metabolic activity (Figure 6b). This drop in metabolic activity could also be linked to cell confluency on day 9.

Taking these results into consideration, CoC scaffolds were prepared, where two different Caf1 proteins were presented on the hydrogels in different spatial distribution. First, the channel was coated with Caf1-YIGSR, then the pore-filled region was coated with Caf1-VEGF (Figure 1c). On co-coating scaffolds, the cell morphology and behaviors significantly altered. Adhered cells inside the channel started to migrate outwards to the pore-filled region. Some cells even exhibited filopodia structure. These results strongly suggest that different spatial presentations of Caf1-VEGF on porous hydrogels drive distinct cell behaviors.

During the last decades, cell–ECM interaction research has shown that when cell-adhesive molecules were spatially presented to the cells in different manners, they induced different patterns of cellular behavior [40,41]. In the case of CoC hydrogels, cell adhesion was achieved thanks to the contribution of the Caf1-YIGSR coating in the channel during the first coating step (Figure 4). The presence of Caf1-YIGSR facilitated proper cell adhesion, where cells could form a strong anchor to the substrate at focal complexes [4]. The presence of Caf1-VEGF promoted protrusion formation of ECs and transformed protrusion into forward movement. This explains the observation of filopodia structure, stress fibers, and polarization of HUVECs seeded on the CoC hydrogels (Figure 7a). The adhered cells sensed migratory signals from the VEGF sequence, which stimulated cell migration processes. In other words, our results suggest that the presence of Caf1-VEGF moieties in the pores created cell directionality, leading to cells moving from the channel outwards to the porous region (exterior of the channel).

### 3.4. Synergistic Effects of Caf1-YIGSR and Caf1-VEGF on EC Morphologies and Behaviors

Taken the outcomes discovered from CoC hydrogels, a question regarding Caf1-YIGSR and Caf1-VEGF spatial distribution on hydrogel was considered. What will be the effect of these two Caf1 proteins on cell morphology and cell behavior, if they were both presented on the hydrogel in similar spatial organization? This question led to the creation of CoCmx hydrogels, where Caf1-YIGSR and Caf1-VEGF solutions were mixed in equal parts (50:50 *v*/*v*) and used to coat the scaffolds via the DFD method. Here, both the channel and the pores were functionalized with Caf1-YIGSR and Caf1-VEGF at the same time. Initial inspection of cell morphology on CoCmx scaffolds showed good cell adhesion (where cells fully lined the channel) and elongated filopodia (which indicated cell sprouting and migration) (Figure 7a, top panel). Moreover, migrating cells connected with non-migrating cells both inside the channel and outside the channel (Figure 7a, bottom panel). These observations suggested the synergistic effect of Caf1-YIGSR and Caf1-VEGF. Both VEGF and YIGSR are known to play a role in angiogenesis, with YIGSR contributing to cell adhesion, cell–cell interactions, and tubule formation, while VEGF stimulates cell migration [4,42,43]. The presence of both Caf1-YIGSR and Caf1-VEGF inside the channel induced a stabilizing adhesive effect on ECs. These ECs then migrated towards the VEGF stimulus that was also available in the pores of the scaffolds. Consequently, the dual presence of YIGSR and VEGF sequences, both exhibiting angiogenic effects, promoted greater EC proliferation. These ECs possibly produced their own ECM, which further stabilized the vessel-like network and induced EC differentiation towards angiogenic phenotypes. This explains why elongated migrating cells were observed in both the channel section and the porous regions outside the channel only on CoCmx scaffolds (Figure 8).

In other words, the dual presence of Caf1-YIGSR and Caf1-VEGF functionalized on our 3D porous hydrogels created a synergistic effect on seeded HUVECs. Previously, a Caf1 mosaic co-polymer containing two pro-osteogenic motifs was seen to promote the early stages of bone formation in primary human mesenchymal stromal cells in a 2D system [35]. The synergistic effect described here further demonstrates the benefits of the Caf1 system, where bioactive peptides can be easily introduced and placed in close proximity in a single material, allowing these synergistic effects to take place. Thus, these effects mimicked the in vitro angiogenesis, where ECs adhered and became activated, then proliferated and differentiated into tip cells, resulting in elongation in the direction of the VEGF stimulus.

### 3.5. Comparison of the Developed Method with Current Vascularization Strategies

Over the past decades, numerous attempts have been made to develop vascularized constructs using three main strategies: microfluidic-based approaches, 3D bioprinting, and organoids/spheroids-based techniques. The readers are invited to read more on this topic in the published review [2].

The use of ECM-based membranes integrated in microfluidic platforms has allowed researchers to develop more physiologically relevant models thanks to the ability to perfuse the systems. However, most models require soft lithography for materials fabrication, which is expensive and is difficult to be used by a wide end-user’s range.

The use of 3D additive manufacturing, such as fuse deposition modeling (FDM), facilitates printing of sacrificial components that better mimic in vivo vasculature. However, these techniques often require several manufacturing steps and still present major issue in terms of resolution. Most vessel geometries remain relatively simple and the vessel diameters are in the range of hundreds of microns. Channels obtained using co-axial bioprinting or with sacrificial bioinks remain in the same range. More recently, the use of laser-assisted bioprinting (LAB) offers high resolution (5–10 µm) of printed channels, automation, reproducibility, and high throughput. Similarly, the use of Vat photopolymerization-based bioprinting opens new possibility to create complex vascular patterns with high precision and high resolution. However, these approaches are still far from translation due to the high cost of equipment, and the need to work with photosensitive materials and photoinitiators further limit their application for therapy.

Spheroids and organoids are another alternative approach to promote the vascularization of hydrogel constructs. They offer the ability to recapitulate the microenvironment, thus present great potential as vascularized models. However, to reach a substantial quantity of tissue, a large number of cells are needed. The use of ECM proteins with heterogeneous composition and high immunogenic potential (e.g., collagen and Matrigel), further prevents translation of these models in the industry and clinical settings.

In this work, we employed a simple method to form microchannels at the microcapillaries range (≥100 µm). Although the filament templating/removal technique is limiting in terms of producing complex designs, it enables high reproducibility and facile fabrication. Our system, porous hydrogels with channels, functionalized in a spatial-controlled manner, present several advantages compared to other aforementioned vascularization strategies.

Compared to other hydrogel-based vascularization strategies, our polysaccharide-based hydrogels support long-term cell culture of up to 9 days, as demonstrated in this study, and could be kept up to 14 days in other studies without being degraded [27]. With a small amount of protein (~0.25–1.8 µg/mg hydrogel), we were able to induce initial cell adhesion, followed by cell proliferation and migration on functionalized scaffolds. Thus, the spatial cues (e.g., YIGSR and VEGF) further direct cell migration mimicking the first step of sprouting angiogenesis. Even though the electrostatic bonds are weaker than covalent bonds, our functionalization method was stable enough to enable observation of grafted Caf1 on the hydrogels (as shown in Figure S5). Moreover, the concomitant presence of channels and pores offers the possibility to promote vascularization of tissue constructs, while enabling co-culture with other cell types for the development of different bioengineered models. Caf1 molecules are manufactured in vitro using bacterial expression systems in high quantities and with a lower cost [33]. Thus, the animal-free origin of Caf1 would reduce immunogenic potential, making them ideal materials for implantable constructs. Our coating method based on ionic interactions is performed in a one-step process and uses green chemistry. In this study we focused on YIGSR and VEGF, but in the future, it will be possible to use the same strategy to incorporate other Caf1 peptides to confer new properties to the material. Finally, from an industrial point of view, our fabrication technique and the choice of materials are highly beneficial: The production method is simple and can be easily scaled-up and the freeze-dried hydrogels allow for long-term storage, all contributing to low-cost production and maintenance.

## 4. Materials and Methods

### 4.1. Materials

Pullulan (Mw 200 kDa) and dextran (Mw 500 kDa) were obtained from Hayashibara Inc. (Okayama, Japan) and Pharmacosmos (Holbaeck, Denmark), respectively. FITC-dextran (dextran labeled with fluorescein isocyanate, TdB consultancy^®^ (Prince George, BC, Canada)) was used to label the hydrogels. All other chemicals were obtained from Sigma-Aldrich^®^ (Saint-Quentin-Fallavier, France). Caf1-YIGSR and Caf1-VEGF as freeze-dried powder were provided to us by Newcastle University (Newcastle, UK).

### 4.2. Hydrogel Synthesis: 3D Porous Polysaccharide-Based Hydrogel with Microchannel

Briefly, a solution of pullulan and dextran (75:25 *w*/*w*) and NaCl was prepared in ultrapure water. This solution is referred to as PUDNA. Then, NaOH 10M was added to the PUDNA solution to activate the hydroxyl groups before reacting with the crosslinker STMP (sodium trimetaphosphate) (3% *w*/*v*) at room temperature under magnetic stirring. The crosslinked solution was poured in between two glass slides, separated by polypropylene suture filaments ø 70 µm (6.0, Ethicon^®^) (Raritan, NJ, USA) and two spacers of 250 µm thickness, before crosslinking in an oven at 50 °C for 20 min. This incubation step was carried out to facilitate the crosslinking reaction and to form microchannels within the hydrogel. Afterwards, the hydrogels were cut into discs of 5 mm in diameter using a biopsy disc-cutter from Harris Uni-Score (Sigma-Aldrich^®^) (Figure 1a).

Hydrogels were neutralized in PBS 10X and washed in distilled water until equilibrium (ca. 15 µS/cm). The conductivity was measured with an Orion 145 A+ conductivity meter purchased from Thermo Fisher Scientific (Asnières-sur-Seine, France). A second wash was performed in NaCl 0.025% (Sigma-Aldrich^®^) until equilibrium (ca. 500 µS/cm). Finally, the hydrogels were freeze-dried to promote pore formation.

The freeze-drying protocol consisted of three stages: freezing under atmospheric pressure from 15 °C to −20 °C at a constant rate of −0.1 °C/min, followed by a phase at constant temperature of −20 °C for 90 min. Primary drying was performed at low pressure (0.001 mbar) and −5 °C for 8 h and secondary drying at 30 °C for 1 h [26].

### 4.3. Hydrogel Characterization

#### 4.3.1. SEM

The topography of freeze-dried hydrogels was observed using the JEOL JSM-IT100 system (software InTouch Scope v.1.060) under low-vacuum conditions. The SEM system was located at the Institute Jacques Monod (Paris, France).

#### 4.3.2. Porosity

The porosity of hydrogels was determined based on a published protocol which calculates the water amount absorbed in the hydrogel before and after manual squeezing tests [44]. Experiments were performed by soaking 5 samples in PBS 1X in a 24-well cell culture plate (Corning^®^) (Corning, NY, USA) for 2 h under mechanical shaking. Samples were then weighed after removing the excess liquid by placing them on the plastic lid. This was considered the weight of the swollen gel (M_swollen_, mg). Following this step, samples were weighed again after squeezing out the remaining liquid using tissue paper and gentle pressing using a spatula. This was considered the “squeezed” weight (M_squeezed_, mg). The porosity calculated by this method corresponds to the large pores that entrap water molecules free or weakly bound to the polysaccharide matrix that are release by gentle mechanical compression. The pore volume percentage was calculated using Equation (1). At least three scaffolds were analyzed per condition. Results were expressed as mean values ± SD.

(1) $$\mathrm{Volume}\;\mathrm{of}\;\mathrm{macropores}\;\left(\%\right)=\frac{\left(M_{\mathrm{swollen}}-M_{\mathrm{squeezed}}\right)}{M_{\mathrm{swollen}}}\times 100$$

#### 4.3.3. Swelling Ratio

Scaffolds were weighed before (M_dry_) and after (M_swollen_) rehydration in PBS 1X for 48 h. The swelling ratio was determined using Equation (2). At least three scaffolds were analyzed per condition. Results were expressed as mean values ± SD.

(2) $$\mathrm{Swelling}\;\mathrm{ratio}=\frac{\left(M_{\mathrm{swollen}}-M_{\mathrm{dry}}\right)}{M_{\mathrm{dry}}}$$

#### 4.3.4. Water Content (WC)

The water content was calculated by using the sample weight after 48 h post-rehydration (M_swollen_) and the sample weights before rehydration (M_dry_). The water content was calculated using Equation (3). At least three scaffolds were analyzed per condition. Results were expressed as mean values ± SD.

(3) $$\mathrm{WC}=\frac{\left(M_{\mathrm{swollen}}-M_{\mathrm{dry}}\right)}{M_{\mathrm{swollen}}}\times 100$$

### 4.4. Hydrogel Functionalization via Electrostatic Interactions

#### 4.4.1. Caf1 Solution Preparation

To assure cell adhesion onto the polysaccharide-based hydrogels, recombinant, engineered Caf1 proteins displaying pro-adhesive and pro-angiogenic peptide motifs were used to functionalize the hydrogels. Briefly, the sequence encoding the peptide was inserted into the *caf1* gene, present on a standard expression plasmid, and the protein was expressed and purified from an *E. coli* culture using tangential flow filtration and size exclusion chromatography [33,35].

The Caf1 proteins with cell-adhesive motifs are called Caf1-YIGSR and Caf1-VEGF. Solutions of 1.0 mg/mL (ɀ = −23.6 mV for Caf1-YIGSR and ɀ = −21.7 mV for Caf1-VEGF) were prepared by diluting the freeze-dried powder in miliQ water at room temperature and stored at −20 °C. These solutions were then thawed on the day of hydrogel coating and allowed to cool to room temperature, before being used.

#### 4.4.2. Cationization of Polysaccharide Hydrogel

Briefly, a predetermined amount of diethylaminoethyl (DEAE)–dextran (Mw 500 kDa) from Pharmacosmos (Holbaeck, Denmark) was added into the standard hydrogel solution to obtain a solution at various concentrations: 5–20% (DD:Dex *w*/*w*; ɀ = +29.5 mV) and mixed well at room temperature (RT) until fully dissolved. The hydrogel precursor solution was degassed overnight at RT and used for hydrogel synthesis the next day.

#### 4.4.3. Spatial-Controlled Hydrogel Coating

To facilitate electrostatic interactions, positive charges were added to the hydrogel network (by incorporation of DEAE–Dextran) to react with the negatively charged protein solution (pI = 4.46). Once the hydrogels were synthesized and rinsed thoroughly (Section 4.1), they were immediately coated via the syringe vacuum-induced method (Figure S1) (100 µL/ gel) and incubated for 2 h at RT. This coating step was performed either only before, or both before and after the freeze-drying step to coat the gel channel only (SFD coating method) or both the channel and pores (DFD coating method) (Figure 1).

### 4.5. Cell Culture and Cell Seeding

Human umbilical vein endothelial cells (HUVECs) (ATCC-CRL-1730) purchased from ATCC^®^ (Manassas, VA, USA) were maintained and subcultured in T75 surface-treated flasks (Corning^®^) in complete endothelial growth medium (EGM-2) (Lonza) following the manufacturer’s recommendations. To prevent bacterial contamination, 1% antibiotic-antimyotic (AA 100X) (Gibco™) purchased from Thermo Fisher Scientific, was also added to the complete growth medium. Cells splitting was performed according to manufacturer and kept in an incubator prior to use (37 °C, 5% CO_2_).

Prior to cell seeding, hydrogels were sterilized under UV light for at least 1 h. Cells were first detached with 1 mL of Trypsin solution (1X, Gibco) at 37 °C for 5 min. Trypsin was inactivated by performing cell dispersion in EGM-2, followed by centrifugation and cell counting. Cell dilution in cell culture medium was conducted to reach the desired concentration. Cell loading was performed via the syringe vacuum-induced method to ensure cell seeding only inside the preformed microchannel. Briefly, hydrogels and cell suspension were introduced in a 10 mL syringe barrel. A 3-way stopcock was used to close the system and the plunger was pulled to make cell suspension circulate inside the channels. Then, cell-loaded hydrogels were placed in a 24 well-plate (Corning^®^), complete cell medium was added, and the plates were placed in an incubator.

The optimal seeding density was determined to be 5.0 × 10^6^ cells/mL. Culture medium (EGM-2) was refreshed every 2–3 days. To facilitate cell lining of the channels, the hydrogels were turned 180° twice following the protocol described in Figure S2.

### 4.6. Cell Metabolic Activity

Cell metabolic activity was determined using the In Vitro Toxicology Assay Kit (Resazurin-based, TOX8-1KT, Sigma-Aldrich, France). Briefly, cells were cultured as previously described (Section 4.3). On day 2, 4, 7, and 10, cell medium was removed and 0.5 mL of fresh culture medium containing 10% resazurin solution was added. After 3 h of incubation (37 °C, 5% CO_2_), 100 μL (in triplicates per sample) of the supernatant was transferred to a 96-well plate. Fluorescence was measured using an Infinite M200 Pro microplate reader (TECAN^®^) at 560Ex/590Em. All samples were analyzed in triplicate, in three different experiments. Results were expressed as mean values ± SD.

### 4.7. Cell Staining for Confocal Microscopy

Cellularized hydrogels were fixed with paraformaldehyde 4% (Sigma-Aldrich^®^) in PBS for 1 h at 4 °C. After rinsing with PBS, membranes were permeabilized with Triton X-100 (Sigma-Aldrich^®^) 0.1% in PBS for 1 h at RT. Actin filaments were labeled by incubation with TRITC-conjugated phalloidin (Sigma-Aldrich^®^) (1/200, 1 h incubation time at RT) and nuclei were stained with DAPI (1/2000). Samples with FITC (λ_ex_ 488 nm) and cellularized samples stained with phalloidin actin marker (λ_ex_ 561 nm) and DAPI nuclear marker (λ_ex_ 405 nm) were observed using a Leica SP8 confocal microscope. Images were acquired using the LSA-X software (LAS X Core 3.7.6) and image analysis was performed with ImageJ/Fiji software (Window, version 153, Java8).

### 4.8. Immunofluoresence Staining of the Caf1 Protein Polymers

In order to confirm the presence of Caf1 protein polymer functionalized on the hydrogel channel (SFD coating method), we conjugated Caf1 with fluorescent markers. Briefly, the primary antibody YPF19 (Yersinia pestis F1 antigen antibody, mouse monoclonal, GTX28275) from GeneTex (Irvine, CA, USA) was prepared in PBS (1/200) to conjugate the Caf1 presented on hydrogels. The functionalized, freeze-dried hydrogels were incubated overnight at 4 °C. After thorough washing in PBS, the samples were incubated with a secondary antibody (Alexa Fluo 647, goat anti-mouse, 1/1000) for 45 min at 37 °C. Finally, the samples were washed in PBS several times for at least 30 min. Then, the samples were observed using CLSM.

## 5. Conclusions

In this study, 3D porous polysaccharide-based hydrogels made of pullulan and dextran that do not promote cell adhesion, were functionalized with animal-free ECM-like molecules via electrostatic interactions promoted by the incorporation of cationized dextran (DEAE–dextran). Although the cationization resulted in slightly opaque samples, we were still able to visualize cell morphology and evaluate in vitro cellular behaviors using 3D microscopy. Our work has demonstrated that electrostatic bonding between the charged hydrogels and Caf1 molecules was stable enough to induce adequate cell adhesion and proliferation. The spatial cues on these scaffolds were controlled through a combination of hydrogel coating and a freeze-drying step. On one hand, ECs adhered and showed sprouting according to how they exposed the cell-adhesive Caf1-YIGSR. On the other hand, the VEGF-like molecule (Caf1-VEGF) functioned as a migratory factor in the presence of the adhesive moiety (Caf1-YIGSR). When ECs were exposed to both Caf1-YIGSR and Caf1-VEGF, they exhibited angiogenic behavior. These results strongly suggest that our functionalized polysaccharide-based hydrogels can provoke different EC behaviors thanks to spatially controlled presentation of these ECM-like, animal-free, pro-angiogenic molecules. Moreover, we also demonstrated that scaffold functionalization via electrostatic interactions was sufficient to promote cell adhesion and cell proliferation for a week, which allowed ECs to further differentiate into their angiogenic phenotypes when exposed properly to the different Caf1 moieties.

The novel approach described here represents an advance in the study of the effect different peptide sequences of the ECM have on ECs behavior. This work represents a proof of concept and opens the door to future studies to determine the effect of other spatial combinations using different Caf1 motifs in different cell types. The pro-angiogenic materials prepared here could be implanted in vivo for regenerative medicine applications. Furthermore, previously in the team, we have demonstrated the formation of soft tissue constructs (e.g., liver spheroids) using the non-functionalized polysaccharide hydrogels [27,45] (Le Guilcher et al., 2022 under revision). These 3D hepatic constructs showed long-term liver functions, including biliary functions, holding promise to be used as 3D models of the liver for theragnostic purposes. The developed polysaccharide hydrogels could be further optimized and integrated with the aforementioned hepatic constructs to build better organ-specific in vitro models. In the near future, we hope to contribute to the translation of vascularized constructs towards clinical applications and drug development.

## Figures and Tables

**Table 1:** Effect of polysaccharide formulation on hydrogel properties: porosity %, swelling ratio, and water content. Results are expressed as mean values ± SD.

Scaffold Name	Porosity %	Swelling Ratio	Water Content (%)
PUDNA	26.1 ± 3.7	13.6 ± 1.8	93.1 ± 0.8
PUDNA-D5	28.0 ± 2.0	14.2 ± 3.7	94.2 ± 0.7
PUDNA-D10	25.0 ± 2.2	10.8 ±1.4	91.5 ± 0.6
PUDNA-D15	25.6 ± 4.3	12.2 ± 2.3	91.9 ± 1.3
PUDNA-D20	25.5 ± 3.1	13.2 ± 2.2	92.8 ± 0.7

## Supplementary Materials

The following supporting information can be downloaded at: https://www.mdpi.com/article/10.3390/ijms232314604/s1, Figure S1: Scheme of syringe coating method which allows to selectively coat only the channel within the hydrogels before pore formation; Figure S2: Cell culture protocol of 5 mm long hydrogel channels. At day 0, endothelial cells (5.0 × 10^3^ cells/μL) were seeded in the channels. Complete endothelial cell culture medium was changed 3 times at day 2, 4 and 6. Hydrogels were turned 180° at day 2 and 4; Figure S3: Shear storage modulus of non-coated hydrogels (NC) and coated hydrogels with different spatial-controlled coating (SgC and CoC); Figure S4: Young’s modulus of hydrogels using nanoindentation mapping; Figure S5: Presence of Caf1-YIGSR on SFD coated hydrogel without cells. Scale bar = 100 µm.
