# Supplementary material for: Spatial-Controlled Coating of Pro-Angiogenic Proteins on 3D Porous Hydrogels Guides Endothelial Cell Behavior
Source: Int J Mol Sci. 2022 Nov 23;23(23):14604. doi: 10.3390/ijms232314604 (PMC9737628; doi:10.3390/ijms232314604)
Supplement: Supplementary file 1 [file ijms-23-14604-s001.zip › ijms-2006233-supplementary.pdf]

Supplementary Materials

Hydrogel coating via syringe vacuum-induced method

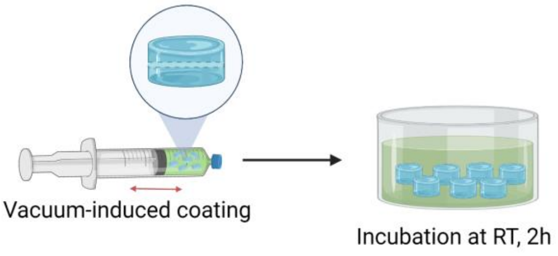

Figure S1. Scheme of syringe coating method which allows to selectively coat only the channel within the hydrogels before pore formation.

Protocol for cell seeding of microchannel within hydrogels

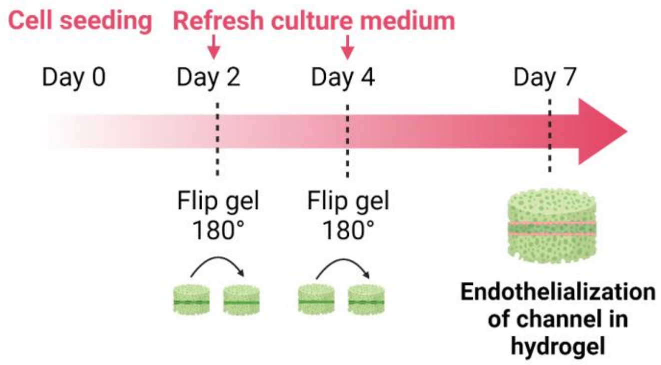

Figure S2. Cell culture protocol of 5 mm-long hydrogel channels. At day 0, endothelial cells ($5.0*10^3$ cells/μL) were seeded in the channels. Complete endothelial cell culture medium was changed 3 times at day 2, 4 and 6. Hydrogels were turned 180° at day 2 and 4.

Bulk mechanical properties

Bulk storage and loss moduli of scaffolds were measured by using the Elastosens™ Bio (Rheolution, Inc., Montreal, QC, Canada). Samples of dimension 22 mm in diameter and 2 mm in height were prepared to fit inside the Elastosens™ Bio sample holder. Samples are were tested in triplicates and results were expressed as mean values ± SD.

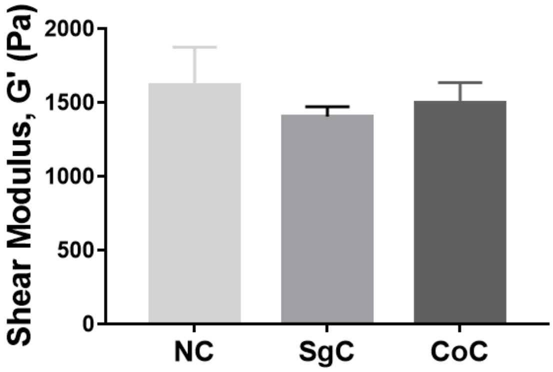

Figure S3. Shear storage modulus of non-coated hydrogels (NC) and coated hydrogels with different spatial controlled coating (SgC and CoC).

Nanoindentation mapping

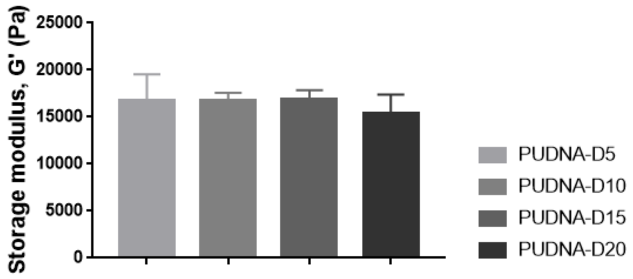

Figure S4. Young's modulus of hydrogels using nanoindentation mapping.

Immunofluorescence staining of the Caf1 protein polymers

In order to confirm the presence of Caf1 protein polymer functionalized on the hydrogel channel (SFD coating method), we conjugated Caf1 with fluorescent markers. Here, we could observe the markers of Caf1 in the channel of the hydrogel (Figure S5). The ablity to observe Caf1 presence on the hydrogel after 2 days of conjugation and rinsage in PBS indicates electrostatic stability. Since the criticial time for cells to adhere and to removel the ECM remains within the first 24 hours after cell seeding, we did not pursue further observation of the functionlized molecules. Plus, this woule require multiple colors in the immunofluoeresence protocol, which will further complicate microscopy obsevation given the sample thickness, opactiy, and presence of cells.

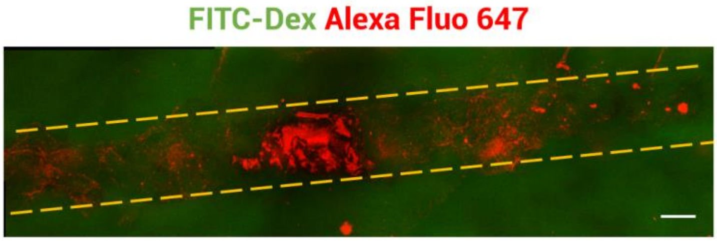

Figure S5. Presence of Caf1-YIGSR on SFD coated hydrogel without cells. Scale bar = 100 μm.
